# The Impact of Vigorous Cycling Exercise on Visual Attention: A Study With the BR8 Wireless Dry EEG System

**DOI:** 10.3389/fnins.2021.621365

**Published:** 2021-02-10

**Authors:** Chin-Teng Lin, Jung-Tai King, Alka Rachel John, Kuan-Chih Huang, Zehong Cao, Yu-Kai Wang

**Affiliations:** ^1^Faculty of Engineering and Information Technology, Australian Artificial Intelligence Institute, University of Technology Sydney, Ultimo, NSW, Australia; ^2^Department of Electrical and Computer Engineering, National Chiao Tung University, Hsinchu, Taiwan; ^3^Brain Research Center, National Chiao Tung University, Hsinchu, Taiwan; ^4^Information and Communication Technology, University of Tasmania, Hobart, TAS, Australia

**Keywords:** dry EEG electrode, P300, vigorous exercise, maximal heart rate, visual attention

## Abstract

Many studies have reported that exercise can influence cognitive performance. But advancing our understanding of the interrelations between psychology and physiology in sports neuroscience requires the study of real-time brain dynamics during exercise in the field. Electroencephalography (EEG) is one of the most powerful brain imaging technologies. However, the limited portability and long preparation time of traditional wet-sensor systems largely limits their use to laboratory settings. Wireless dry-sensor systems are emerging with much greater potential for practical application in sports. Hence, in this paper, we use the BR8 wireless dry-sensor EEG system to measure P300 brain dynamics while cycling at various intensities. The preparation time was mostly less than 2 min as BR8 system’s dry sensors were able to attain the required skin-sensor interface impedance, enabling its operation without any skin preparation or application of conductive gel. Ten participants performed four sessions of a 3 min rapid serial visual presentation (RSVP) task while resting and while cycling. These four sessions were pre-CE (RSVP only), low-CE (RSVP in 40–50% of max heart rate), vigorous-CE (RSVP in 71–85% of max heart rate) and post-CE (RSVP only). The recorded brain signals demonstrate that the P300 amplitudes, observed at the Pz channel, for the target and non-target responses were significantly different in all four sessions. The results also show decreased reaction times to the visual attention task during vigorous exercise, enriching our understanding of the ways in which exercise can enhance cognitive performance. Even though only a single channel was evaluated in this study, the quality and reliability of the measurement using these dry sensor-based EEG systems is clearly demonstrated by our results. Further, the smooth implementation of the experiment with a dry system and the success of the data analysis demonstrate that wireless dry EEG devices can open avenues for real-time measurement of cognitive functions in athletes outside the laboratory.

## Introduction

The current thinking in sports neuroscience is that athletic performance can be improved by developing a winning brain. Definitive proof, however, demands better understandings of the links between the brain and physical behavior and some innovative biometric measurement tools (Park et al., 2015). In this regard, brain imaging is providing a new approach to training by revealing deeper insights into the interrelations between psychology and physiology in sports science. Among all the brain imaging techniques, electroencephalography (EEG) is one of the most powerful. It has a higher temporal resolution than fMRI or MEG, and it also costs less. EEG measurements use the time and/or frequency dynamics of electrical activity in the brain to infer what types of cognitive processes are taking place. As such, they have been used extensively to explain a person’s brain state during sport and exercise (Cheron et al., 2016; Perrey and Besson, 2018). For example, EEG indexes have been used to study: the differences in brain activity between champions and novices (Del Percio et al., 2008; Babiloni et al., 2010; Cheng et al., 2015; Wang and Tu, 2017; Wang et al., 2019) and to maintain optimal sporting performance through neurofeedback training (NFT) (Cheng et al., 2015; Mirifar et al., 2017; Xiang et al., 2018).

Traditional wet sensor-based EEG systems, i.e., “wet, wired systems” provide excellent signal quality but require proper skin preparation with the application of conductive gel in order to minimize impedance at the skin and sensor interface (Miller and Harrison, 1974). So, the preparation time is usually long and a bit messy as these conductive gel leaves residue on the scalp. Moreover, the skin preparation for use of wet electrodes is generally uncomfortable for participants as the repeated gel application poses an infection risk and occasionally, the procedure might be painful due to skin abrasions. Furthermore, there might be short circuit between electrodes if the gel leaks out of the electrode (Merletti, 2010). Additionally, these traditional wet sensor-based EEG systems are particularly not suitable for long-term studies as the signal quality may deteriorate over time as the gel dries (Ferree et al., 2001). Another major issue with the traditional EEG systems is that the wired connections from the electrodes need go to the amplifier, which greatly limits the mobility of the participants, hindering the deployment of these wired EEG systems in real-world applications. For sports science, this severely limits the practical applications of EEG analytics (Park et al., 2015; Bertollo et al., 2019; Wang et al., 2019). To overcome this constraint, several dry, wireless systems have been developed over the past decade (Liao et al., 2011; Chen et al., 2014; Lin et al., 2014, 2019, 2020; Lopez-Gordo et al., 2014). These systems bring great convenience and portability that could help brain imaging progress from lab-based research to field applications (Lopez-Gordo et al., 2014; Xu et al., 2017). Compared to the fitting and removal of wet electrodes, which require specific expertise and time (Chen et al., 2014), the easy-to-handle dry electrodes can be quite helpful in fostering the practical application of EEG in sports while minimizing variances related to measurement errors (e.g., cross-talk between electrodes due to excessive amounts of gel application).

The reliability of dry systems in measuring brain dynamics has already been demonstrated with real-world brain-computer interfaces (Lin et al., 2019), education (Xu and Zhong, 2018), and clinical assessment (Lin et al., 2017). However, their use in sports science has barely been explored. Recently, di Fronso et al. (2019) used an endurance cycling task to compare the performance of a novel 64-channel dry sensor cap versus a gel-based one. Their results show the average preparation time of the dry cap at around a third that of the gel-based cap. Further, through an average power spectral density analysis, they found the quality of measurements from both systems to be equivalent. Bertollo et al. (2017) reach the same conclusion in a study of individual alpha peak frequencies (iAPFs) induced by fatigue (Bertollo et al., 2017). Dry electrode sensors have also been shown to reliably detect the P300 component of event-related potentials (Zander et al., 2011; Mathewson et al., 2017).

Since attention promotes goal-directed behavior by reducing distractions from external stimuli, it is a critical cognitive ability during exercise and competition (Furley and Wood, 2016). P300 waves are a reliable indicator of attention (Polich, 2007; Wang et al., 2016; Kao et al., 2020), and some studies have shown this brain dynamic has strong links to sports expertise (Nakata et al., 2010; Zhang et al., 2015; Wang and Tu, 2017), although the correlations are far from conclusive. Further, there is still no consensus on its effects (McMorris and Hale, 2012). Magnié-Mauro et al. (2000), for example, use significant increases in P300 amplitude and significant decreases in P300 latency to demonstrate that long-term exercise has a favorable effect on cognitive functions, whereas Killane et al. (2013) show both remain stable across different exercise conditions. Their study used a conventional wet sensor-based EEG system to investigate the amplitude and latency of P3 event-related potentials in an auditory oddball task while subjects were seated (static condition) and while cycling (in place) (dynamic condition). Using a small, mobile EEG system, Debener et al. (2012) obtained reliable P300 readings during an auditory oddball paradigm where participants walked outdoors around a university. Their results show that single-trial P300 measurements can be categorized, with a further correlation analysis showing a strong association between P300 amplitudes in indoor and outdoor recording conditions. Zink et al. (2016) used a mobile EEG system to evaluate the ERP characteristics of a three-class auditory oddball paradigm. The scenarios were sitting still on a bike, pedaling a fixed biked and biking freely around – all outdoors. They noted a decrease in P300 amplitude in the free biking condition as compared to the still and fixed pedaling condition. Using an ICA approach, Gramann et al. (2010) contrasted ERPs recorded using standard equipment during a visual oddball task with four movement conditions: performed on a treadmill, standing, slow walking, fast walking and running. Their results provide evidence of comparable P300 effects across different movement conditions, demonstrating that reliable effects can be obtained during moderate whole-body movements. However, their study is limited to a laboratory setup.

Wireless dry-sensor EEG devices are a critical part of bringing BCI applications to the real world. In this study, we explore the impact of exercise at varying intensities on visual attention using a wireless dry-sensor EEG system (Lin et al., 2011, 2015). Our results make several contributions to the literature:

• evidence that dry EEG systems can reliably assess the brain dynamics during exercise
• the effect of varying intensities of exercise on visual attention.

## Materials and Methods

### The EEG Cap and Dry Sensors

**FIGURE 1:**
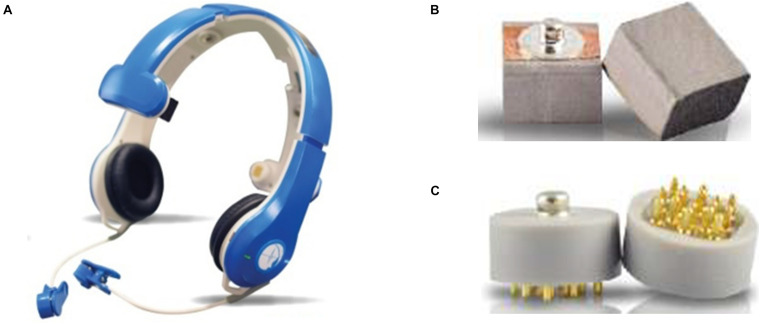
**(A)** The BR8 EEG acquisition device; **(B)** a foam sensor; and **(C)** a spring-loaded sensor, both used in the BR8 system.

The EEG data were collected using the BR8 system (Brain Rhythm Inc.), designed by our research team. This EEG device (Figure 1A) uses two types of dry sensors, Foam-based and Spring-loaded dry sensors (Figures 1B,C). The foam sensors are made of an electrically conductive polymer covered in a conductive fabric. The spring-loaded sensors have eight probes coated in gold arranged in a geometric configuration that establishes good electrical contact with an irregular scalp surface with low skin-electrode interface impedance. To avoid any pain should force be applied to the sensors, the bottom surface is covered with rubber padding. Both types of sensors can measure EEG signals without any conductive gel. Detailed specifications for each are shown in Table 1.

**TABLE 1:** Specifications of the dry sensors in the BR8 wireless EEG acquisition system.

	Foam	Spring-loaded
Size (mm)	15×15×14	15×15×14
Weight	0.8g	1.8 g
Impedance	200–500 K	200–500 K
Average life span	60 h	8,760 h
Position	No hair area	Hairy area

The performance and reliability of the BR8 system is comparable to conventional wet sensor-based systems (Liao et al., 2014). And the efficacy of both types of sensors has been validated in a series of studies: Liao et al., 2011, 2014, Lin et al., 2011, 2019, 2020, and Yu et al., 2016. Foam based sensors are used on the hairless region (Lin et al., 2011), therefore mainly in the frontal electrodes, and spring-based sensors are used for the remaining electrodes as hairs reduces the contact area at the electrode-skin interface (Liao et al., 2014). Of course, the main benefit is that the cap takes considerably less time to fit since there is no need for gel. The EEG channel locations follow the international 10–20 system and include FP1, FP2, Fz, C3, C4, Pz, O1, and O2 with average impedance values below 210 KΩ. In this study, we collected data from all eight channels, which cover most areas of human brain including frontal, central and posterior regions. Only the signals in the Pz channel were used to analyze P300 wave as the traditionally maximum P300 amplitude is observed in this posterior region (Polich, 2007). P300 waves are known to reflect attention and memory processes (Isreal et al., 1980; Wickens et al., 1983). Since we applied RSVP experimental procedure with visual stimulus in this study, the P300 amplitude in the Pz channel will be the largest (Rosenfeld et al., 1999; Gonsalvez et al., 2007; Polich, 2007). Therefore, only the Pz channel was used for further P300 wave analytics.

### Participants

Ten male college students, at an average age of 23.5 ± 1.5 years, were recruited from National Chiao Tung University. All participants reported corrected-to-normal vision and no history of neurological or heart problems. Additionally, all signed a consent form before performing the experiment. Ethical approval was obtained from the Research Ethics Committee for Human Subject Protection of National Chiao Tung University (NCTU-REC-106-057).

### Experimental Procedure

**FIGURE 2:**
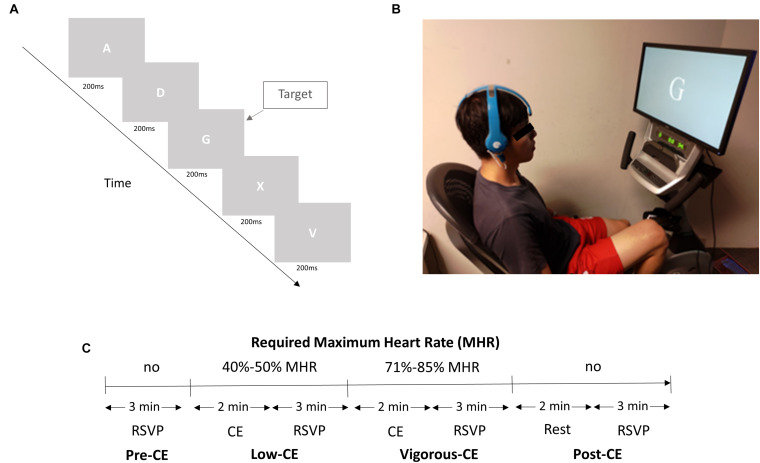
**(A)** The experiment setup. **(B)** A subject performs the RSVP task and cycling exercise simultaneously wearing the BR8 wireless EEG system. **(C)** Experiment design with four sessions. The different exercise intensities are set as a percentage of the participant’s estimated maximum heart rate (Loprinzi and Kane, 2015).

The experiment consisted of a rapid serial visual presentation (RSVP) task to be performed four times, once with a resting heart rate, once after low-intensity exercise, once after high-intensity exercise, and a fourth and final time after a 2 min rest post vigorous exercise. The salient details are illustrated in Figure 2A. Specifically, the task required participants to watch a screen, which displayed a random letter of the alphabet in white against a gray background every 200 ms, equating to five visual stimuli per second (5 Hz) (see Figure 2A). One letter was chosen as the target letter, which was presented randomly in a stream of distractor letters. The participants were asked to left click a wireless mouse as soon as they identify the target letter. The rate of occurrence of the target was approximately 5% and the targets were randomly distributed in each 3 min RSVP session. Each target presentation was separated from the preceding target presentation by at least 900 ms. In the first of the four sessions [pre-CE (cycling exercise)], the participants simply performed the RSVP task without cycling. However, in the next two sessions, the participants were required to cycle for 2 min at a specific percentage of their estimated maximum heart rate [HR(max)] before performing the task (Loprinzi and Kane, 2015; Figure 2C). Proposed by the American Heart Association, a person’s HR(max) is calculated according to a standard equation, MHR = 220 − age ^∗^ (0.7∼0.8). The cycling was performed on a stationary mechanical recumbent cycle ergometer (Matrix R1x, Johnson Inc.), as shown in Figure 2B. To help them reach the required heart rate, the ergometer was automatically increased by 20 W every 30 s until they were within the specified HR(max) range. The range for the second low-CE session was 40–50% of their HR(max), and 71–85% for the third vigorous-CE session. In the fourth and last session, i.e., post-CE, the participants rested for 2 min and then performed the RSVP tasks for a final time.

Prior to the actual experiment, the participants were fitted with a wireless EEG acquisition system (Figure 2B) with dry sensors (Brain Rhythm Inc.) and heart rate band (Bioharness 3 monitor, Zephyr Technology Corporation) in a soundproof lab setting. The device was fixed in place using a bandage to maintain electrode-skin contact stability. The participants were instructed to avoid head movement and electrode impedance was monitored continuously. As the sensors have a flexible substrate, it allows for high conformity between the irregular scalp surface and the electrode, thereby, molding well to the scalp surface to achieve and maintain stable electrode-skin contact. The BR8 headset is lightweight, with an unobtrusive design that provides great wearing comfort (Lin et al., 2010; Liao et al., 2011; Radüntz and Meffert, 2019). The experimenter also evaluated and confirmed with each participant the wearing comfort of the BR8 headset before the start of each session. The preparation time was less than 2 min as the sensors were able to attain the required skin-sensor interface impedance for its operation. The average impedance at the foam-based sensors was 103 ± 10 KΩ and at the spring-based sensor, it was 85 ± 7 KΩ. The ground and reference electrodes, both foam-based sensors, are applied with a clip on each ear lobe. Additionally, the participants were asked to practice both the RSVP task and cycling exercise. The whole experiment was designed in such a way as to hold the intensity of the exercise, i.e., the subject’s heart rate, at a consistent level during each session. The participant’s heart rate, displayed on a monitor, was continuously monitored by the experimenter throughout the experiment. Participants were required to increase the speed of cycling exercise to achieve the required heart rate for a specific session. The experimenter instructed the participants to adjust the speed of cycling exercise to keep their heart rate stable for a specific session. No resting interval was set, and the total duration of the experiment was 20 min.

### Behavioral Data Analysis

Accuracy and reaction times were recorded as the behavioral data. Reaction time (RT) was defined as the time from the appearance of the target to the mouse click. Accuracy was calculated as the ratio of correct detections, both target and non-target, to the total number of corresponding appearances. For example, in a session where 847 letters appeared that were not the target letter, non-target detection accuracy was calculated as the number of times the participant *did not* click the mouse, divided by 847. SPSS was used for the statistical analysis.

### EEG Data Analysis

The EEG data were pre-processed and analyzed using the MATLAB toolbox, EEGLAB (Delorme and Makeig, 2004). Each epoch included the signal from 100 ms before the target appearance to 800 ms afterward. Each epoch was baseline corrected using the average EEG activity in the 100 ms before the target onset. The epochs with incorrect responses were not considered for P300 analytics. To investigate the brain dynamics elicited with high signal-to-noise ratios (SNR) and to estimate the P300 component, ensemble averaging (EA) was applied to all epochs. Ensemble averaging method involves averaging all the target trials to extract the event related potential from EEG background activity (Luck, 2014) and is a commonly employed method to reliably detect and amplify the P300 wave (Rakotomamonjy and Guigue, 2008; Mak et al., 2011; Shi et al., 2012; Bekdash et al., 2015; Vareka and Mautner, 2015). During preliminary analysis, we evaluated the amplitude of P300 wave in the C3, C4, and Pz channels, however, the maximum amplitude was observed in the Pz channel as reported widely in the literature (Polich, 2007; Lin et al., 2015). Therefore, we evaluated the P300 amplitude in the Pz channel at around 250–500 ms across all experimental sessions.

To eliminate interference, we followed the difference wave method (target minus non-target) and all points within the range were averaged for further comparison. Statistical differences in the heart rate and P300 amplitude comparisons were identified through two-tailed paired *t*-tests with the *p*-values adjusted according to the false discovery rate (FDR).

## Results

### Heart Rate

**FIGURE 3:**
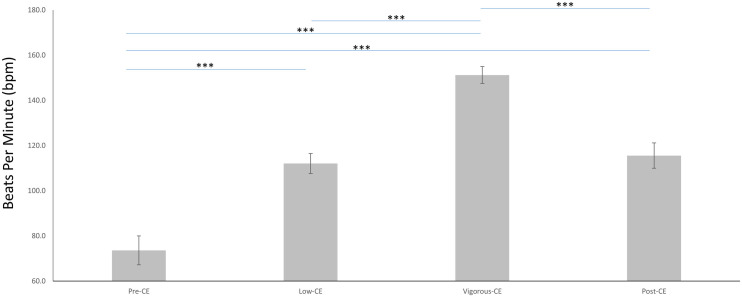
Average heart rates (in bpm) during the four experimental sessions (Pre-CE, Low-CE, Vigorous-CE, and Post-CE). ^∗∗∗^*p* < 0.001.

The mean heart rates for the four sessions are shown in Figure 3 and listed as follows: pre-CE mean 73.6 ± 6.4 bpm; low-CE mean 112.0 ± 4.4 bpm; vigorous-CE mean 151.2 ± 3.7 bpm; and post-CE mean 115.5 ± 5.6 bpm. The paired *t*-tests with FDR correction was employed to compare the heart rates during different sessions. Unsurprisingly, the pre-CE heart rates were significantly lower than both the low-CE rates [*t*(9) = 17.69, *p* < 0.001] and the post-CE rates [*t*(9) = 20.21, *p* < 0.001]. And the vigorous-CE rates were significantly higher than all other sessions at: pre-CE *t*(9) = 43.28, *p* < 0.001; low-CE *t*(9) = 25.56, *p* < 0.001; and post-CE *t*(9) = 38.87, *p* < 0.001. There was little difference between the heart rates for the low- and post-CE sessions [*t*(9) = 2.09, *p* = 0.066].

### Behavior

**FIGURE 4:**
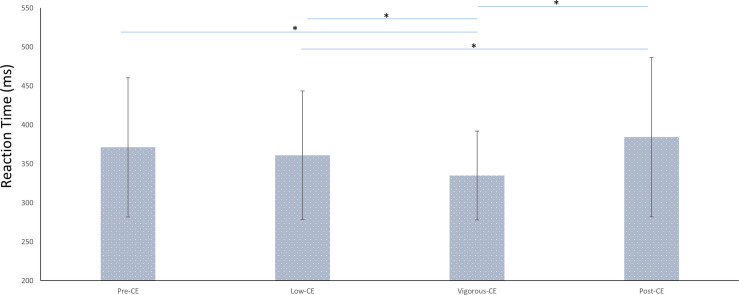
Average reaction times (in ms) in different sessions (with different maximal heart rate conditions). ^∗^*p* < 0.05.

These behavioral results indicate the impact of different intensities of exercise on the cognitive performance. Figure 4 shows the average reaction times (RT) for each session. The average reaction time for each session were: pre-CE mean 371 ± 89 ms; low-CE mean 361 ± 82 ms; vigorous-CE mean 335 ± 56 ms; and post-CE mean 384 ± 102 ms. The paired *t*-tests with FDR correction show that participants responded significantly faster to the targets in the vigorous-CE session than in the pre-CE [*t*(9) = −2.76, *p* = 0.022], low-CE[*t*(9) = −2.63, *p* = 0.027], and post-CE session [*t*(9) = −3.02, *p* = 0.014]. Further, RTs in the post-CE session were significantly greater than that of the low-CE session [*t*(9) = 2.42, *p* = 0.038], but not the pre-CE session [*t*(9) = 1.971, *p* = 0.08]. There was no difference [*t*(9) = 1.56, *p* = 0.152] between the RTs for the pre-CE and low-CE sessions. These results show that exercise enhances attention.

**FIGURE 5:**
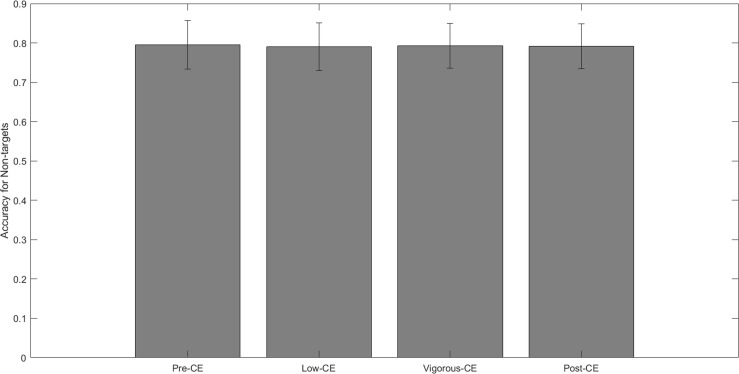
Average non-target detection given different maximal heart rate conditions.

**FIGURE 6:**
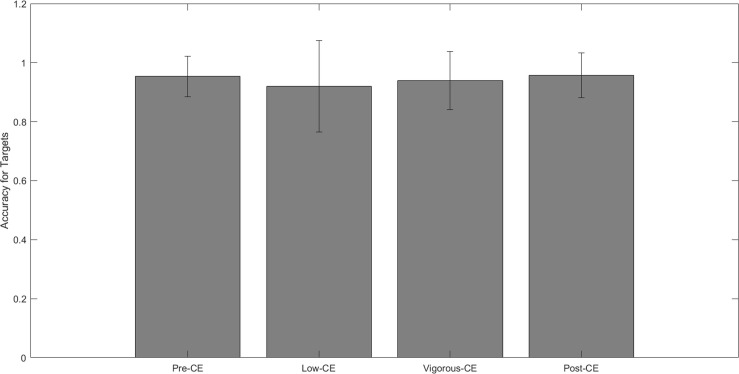
Average target detection given different maximal heart rate conditions.

Figure 5 shows the mean accuracy of correctly identifying non-targets for each session, i.e., *not* clicking the mouse when the displayed letter is not the target. The mean accuracy for each session is: pre-CE mean 0.79 ± 0.06; low-CE mean 0.79 ± 0.06; vigorous-CE mean 0.79 ± 0.05; and post-CE mean 0.79 ± 0.05. The paired *t*-tests with FDR correction reveal no significant differences between these accuracy rates. Figure 6 shows the mean accuracy for the targets at: pre-CE mean 0.95 ± 0.06; low-CE mean 0.91 ± 0.15; vigorous-CE mean 0.93 ± 0.09); and post-CE mean 0.95 ± 0.07. The low-CE session had the lowest accuracy, but the differences were not statistically significant (pre-CE session *t*(9) = 0.925, *p* = 0.189; vigorous-CE *t*(9) = 0.741, *p* = 0.239; post-CE session *t*(9) = 1.439, *p* = 0.09. Therefore, accuracy did not vary significantly across the different sessions.

### P300 at Different Maximal Heart Rates

**FIGURE 7:**
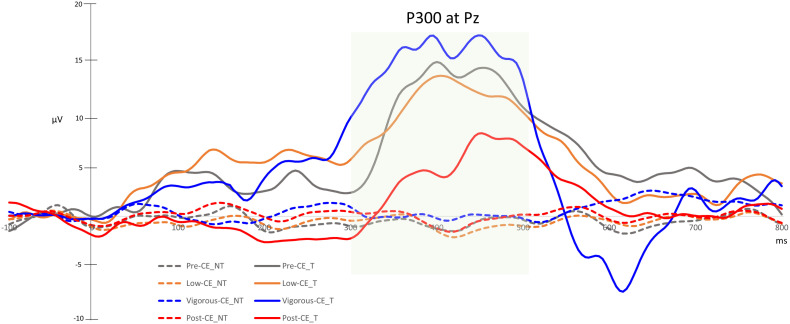
Averaged P300 values for target (T) and non-target (NT) detection in the Pz channel.

P300 waves reflect brain dynamics involved with decision making, particularly stimulus evaluation and categorization. Hence, these results indicate visual attention. Figure 7 shows the ERP waveforms recorded in the Pz channel during each of the sessions. Table 2 summarizes the P300 amplitudes for the target (T) and non-target (NT) and the difference wave generated by subtracting the non-targets from the targets. The paired *t*-tests with FDR correction show a significant difference between the target and non-target during all sessions. The P300 component for target detection was clearly evoked at different levels of exercise. The amplitude of P300 in the post-CE session was significantly lower than in the other sessions: pre-CE *t*(9) = −2.57, *p* = 0.030; low-CE *t*(9) = 2.51, *p* = 0.033; and vigorous-CE *t*(9) = 4.10, *p* = 0.003. Although the amplitude for the vigorous-CE seems notably higher, the differences to the other sessions were not significant: pre-CE and low-CE *t*(9) = 0.26, *p* = 0.805; pre-CE and vigorous-CE *t*(9) = 0.50, *p* = 0.629; and low-CE and vigorous-CE *t*(9) = −1.84, *p* = 0.099. There was a marked declining trend in P300 amplitude during the vigorous-CE session.

**TABLE 2:** The mean ± SD for P300 amplitude in the Pz channel with the wireless EEG system for different heart rate conditions.

	Target	Non-target	*t*-value	*p*-value	Difference wave (target-non-target)
Pre-CE	12.90 (9.76)*	−0.60 (1.38)*	4.75	<0.001	13.50 (8.89)*
Low-CE	11.58 (6.79)*	−0.98 (1.35)*	4.93	<0.001	12.57 (8.07)*
Vigorous-CE	15.11 (7.17)*	−0.10 (1.38)*	7.88	<0.001	15.2 (6.10)*
Post-CE	5.19 (2.03)*	−0.28 (0.93)*	8.78	<0.001	5.47 (1.97)*

*SD are in parentheses.

## Discussion

In line with others that have found exercise influences cognitive performance (Tomporowski et al., 2005; Sibley et al., 2006; Chang et al., 2011; Voss et al., 2011; Chaddock et al., 2012; Vestberg et al., 2012, 2017; Cona et al., 2015), working memory (Komiyama et al., 2015), and executive function (Chang et al., 2015), our results show considerable evidence that exercise enhances cognitive performance.

The experimental task was designed in a manner to evaluate the visual attention during a cycling task that mimics real-world exercise condition. Consistent with a realistic exercise condition, initially the participants are at rest before starting the exercise at a low-intensity and then proceeding to a more vigorous exercise at the end. Therefore, the participants performed RSVP task before performing any cycling exercise in the first session. In the second session, the participants performed RSVP task with a low-intensity exercise. Further, the third session reflects the attention in maximal exercise condition where the participants perform RSVP task in vigorous exercise. The fourth session reflects the cool-down condition after exercise where the attention of the participants after performing exercise is evaluated. The sequence of cycling exercise was not randomized as it might be strenuous for participants to perform vigorous exercise swiftly from the rest condition(s). Therefore, we designed the task such that the participants can gradually increase the intensity of the exercise, mimicking realistic exercise conditions. However, the intensity of exercise, evaluated by the heart rate of the participant, is held at a consistent level in each session. Thus, the observed effects in reaction time and P300 amplitude during each session cannot be explained by cross-condition influences.

In this study, we examined the impact of exercise on visual attention using a classical RSVP task. The randomization of single-target trials in each session diminished response biases related to expectancy. However, our results show high level of accuracy in identifying the target in all the four sessions. This can be explained by our RSVP task design as it is widely reported in the literature that accuracy is high in a single-target RSVP task (Raymond et al., 1992). In our RSVP task, we found that the participants had enhanced attention and responded significantly faster after performing vigorous cycling exercise.

It has been demonstrated in many studies that exercise also induces a higher P300 amplitude (Tomporowski and Ellis, 1986; Magnié-Mauro et al., 2000; Hillman et al., 2003; Kamijo et al., 2007; Won et al., 2017), which suggests that vigorous exercise is of value to cognitive performance, particularly, attention. P300 amplitude is known to be proportional to the number of attentional resources allocated for a particular task (Wickens et al., 1983; Schubert et al., 1998; Kida et al., 2004) and an indication of the allocation of attention and context updating in working memory (Donchin and Coles, 1988). In our tests, P300 amplitudes were higher during vigorous-CE (Ref. Figure 7). However, P300 amplitudes for the post-CE session were significantly lower than that of the other sessions, probably as a result of peripheral and central fatigue (Faria et al., 2005; Dempset et al., 2006). Our findings show that vigorous exercise enables attentional resource allocation, and more resources were allocated to the RSVP task during the vigorous-CE session, suggesting the value that vigorous exercise brings to executive control function.

Increased P300 amplitudes following intense exercise is explained to be the result of general arousal and enhanced attention (Magnié-Mauro et al., 2000). Higashima et al. (1996) found a direct correlation between increased P300 amplitudes during vigorous exercise and increases in cerebral blood flow. Further, according to Bhambhani et al. (2007), cerebral blood flow increases during exercise until the subject reaches a respiratory compensation threshold and then decreases due to fatigue, explaining the decreased P300 amplitude during post-CE session. Our findings also indicate an increased P300 amplitude during vigorous exercise, which confirm that the observed increase in the P300 amplitude is a function of the intensity of the exercise. Another major highlight of our work is the use of the BR8 dry sensor EEG system, which greatly reduced the setup time as no skin preparation was required. Studies with oddball experiments both stationery (Zander et al., 2011) and walking (Debener et al., 2012; De Vos et al., 2014) have already demonstrated that dry sensor systems can measure P300 amplitudes as reliably as conventional wet sensor systems. Moreover, the performance and reliability of the signals recorded by the BR8 was demonstrated to be comparable to traditional wet sensor-based systems (Liao et al., 2014). Furthermore, in our earlier study (Lin et al., 2015), the BR8 system demonstrated great success in detecting P300 waves. In current study, we evaluated the amplitude of P300 wave in the Pz channel as the maximum amplitude is reported to be observed in the Pz channel (Polich, 2007; Lin et al., 2015). However, despite recording the signals in eight channels, it is only due to the nature of the experiment that we limited our analysis to Pz channel. Even though only a single channel was evaluated in this study, the quality and reliability of the measurement using these dry sensor-based EEG systems is clearly demonstrated by our results.

However, the EEG signals during a real-world sports activity might be plagued with various artifacts due to sports movement, including electromyography artifacts from muscular activity and ballistocardiographic artifacts (Thompson et al., 2008; Gwin et al., 2010; Kline et al., 2015). Therefore, more rigorous artifact removal strategies should be employed in such real-world sport applications (Gramann et al., 2014; Cao et al., 2015). Moreover, BR8 system can be conveniently deployed in mobile real-world scenarios as it is wireless and transfers data via Bluetooth as compared to traditional EEG devices that require the wired connections to go from the electrodes to the amplifier. However, deploying the device in long term EEG studies extending over 2 h might be limited as the device battery will need charging.

Our findings in the RSVP task enrich the literature with evidence that dry sensor-based EEG systems can reliably detect P300 waves and amplitudes. As P300 waves reflect changes in attention, this study shows that it is possible to measure attentional resources using dry sensor EEG systems. In the current study, we measured event-related potential and the effect of exercise on visual attention, but there are many other forms of attention that could and should be explored. Further, similar studies with a greater number of subjects would add strength to these findings. Nevertheless, our results are encouraging and pave the way for a closed-loop BCI system with wireless, dry sensor-based EEG systems in real-world sports environments.

## Conclusion

In this study, we show that it is possible to measure brain dynamics accurately and reliably during a cycling exercise using dry-sensor EEG systems. Furthermore, we demonstrate that exercise improves visual attention, as evidenced by decreased reaction times and increased P300 amplitudes. Beyond providing evidence that EEG activity can indicate intensity of exercise and attention levels, our results also demonstrate that dry EEG systems have great potential for convenient, real-time monitoring of athletic performance through brain dynamics in the field in the near future. Future research should explore the use of dry sensor systems in field settings with a greater number of subjects to gain more accurate and statistically significant results. However, these promising results so far should promote investigation of attentional cues in real-world settings, thereby advancing sports neuroscience.

## Data Availability Statement

The raw data supporting the conclusions of this article will be made available by the authors, without undue reservation.

## Ethics Statement

The studies involving human participants were reviewed and approved by the Research Ethics Committee for Human Subject Protection of the National Chiao Tung University (NCTU-REC-106-057). The patients/participants provided their written informed consent to participate in this study.

## Author Contributions

All authors have contributed significantly to the work, have read the manuscript, attest to the validity and legitimacy of the data and its interpretation, and agreed to its submission to Frontiers in Neuroscience.

## Conflict of Interest

The authors declare that the research was conducted in the absence of any commercial or financial relationships that could be construed as a potential conflict of interest. The reviewer L-DL declared a past co-authorship with several of the authors C-TL, J-TK, and Y-KW to the handling editor.
